# Leveraging public engagement to improve healthcare quality: The role of community and stakeholder engagement in Colombia’s National Quality of Care Strategy

**DOI:** 10.1371/journal.pgph.0005333

**Published:** 2025-11-04

**Authors:** Kayla A. Benjamin, Breanna K. Wodnik, Emily Cordeaux, Laleh Rashidian, María Isabel Riachi, Lauren E. Smith, Mariana Morales Vazquez, Jeremy Veillard, Beverley M. Essue

**Affiliations:** 1 Institute of Health Policy, Management & Evaluation, University of Toronto, Toronto, Ontario, Canada; 2 World Bank Group, Bogotá, Distrito Capital, Colombia; School of Public Health, College of Health Science, Addis Ababa University, ETHIOPIA

## Abstract

Public engagement, also referred to as community and stakeholder engagement (CSE), and high-quality care are core components of strategies to achieve universal health coverage (UHC), one of the United Nations Sustainable Development Goals. As part of the movement toward achieving UHC, Colombia has developed one of the first national quality of care strategies in the Latin America and Caribbean region. However, the degree to which public engagement was considered in the development and implementation of Colombia’s National Quality of Care Strategy (the Strategy) is not clearly understood. With a growing global consensus on the importance of public engagement in health systems and policy, we use a qualitative case study comprising a document analysis followed by qualitative interviews, to explore how CSE has been considered in the design and implementation of the Strategy. In an analysis guided by the Lavery framework for CSE, we address the following three research objectives: 1) describe how community and stakeholder engagement is reflected in the Strategy; 2) explore approaches undertaken to engage community and stakeholders in the development and implementation of the Strategy and their perceived effectiveness; and 3) report on strengths and opportunities for improving CSE in health policymaking in Colombia. Our findings demonstrate a strong written commitment to CSE. However, the implementation of engagement strategies fell short in including community (i.e., patients and citizens), particularly those from structurally marginalized communities, due in part to inconsistent political and financial support and the absence of evaluation mechanisms. These findings have important implications for Colombia and other comparable jurisdictions aiming to enhance public engagement in health policy making. Our study highlights the need to move beyond symbolic participation toward inclusive, well-resourced, and systematically evaluated engagement strategies.

## Introduction

There is a growing consensus on the importance of public engagement in health systems and policy in low- and middle-income countries (LMIC), with unprecedented opportunities for public engagement in higher-level policy and program decisions [1–4]. While encouraging, examples of governments meaningfully incorporating public perspectives into health system decision-making remain scarce [5]. Public engagement activities undertaken by governments remain predominantly focused on soliciting feedback on quality of health services or research projects, rather than higher-level policy decisions [6]. In a Cochrane systematic review of 7,272 governance arrangements for health systems in LMICs, no studies evaluated the effects of public and community participation in organisational or policy decisions [7]. When the public is engaged in health policy, it is often tokenistic and does not result in meaningful engagement in decision-making [6]. As outlined by Lavery and colleagues, meaningful and effective engagement includes, among other elements, early initiation, informed participation, relationship building, dedicated resources, and evaluation of engagement strategies [8].

This paper presents a case study of Colombia’s updated National Quality of Care Strategy (2022–2027) (herein referred to as ‘the Strategy’). The Strategy was developed following the formal evaluation of the earlier version covering 2016–2021 by the World Bank Group, which included specific recommendations to strengthen public engagement [9,10]. We explore how public engagement has been considered in the design and implementation of the Strategy, assess the impact of engagement approaches, and identify opportunities for improvement. As such, this paper offers valuable insights for Colombia in strengthening engagement practices in high-level policy and program decision-making, as well as for other comparable jurisdictions aiming to meaningfully integrate public perspectives into health system governance.

### What do we mean by public engagement?

Public engagement is a loose concept, often used interchangeably with public participation, public consultation, community engagement, and citizen engagement. Researchers often set parameters as to *who* constitutes the ‘public’ or *which* mechanism(s) of engagement are used as ways of focusing the scope of public engagement. Across 175 articles, Mitton and colleagues identified 15 different public engagement techniques (e.g., public hearing, opinion poll, consultation document) and three distinct categories of ‘public’ (patients or service users, representatives of organised groups, and individual citizens) [3].

In alignment with this paper’s guiding framework (detailed below) [11], we use “community and stakeholder engagement” (CSE) to specifically outline public engagement. For our paper, we adopt the concept of “community and stakeholder,” and we include *all* individuals, groups or organisations that may be affected by the Strategy. For example, community would include patients, service users, and individual citizens affected by the quality of care in Colombia, while stakeholders would include those responsible for implementing the strategy, such as healthcare practitioners and public servants. We do not focus the scope of CSE on specific mechanisms of engagement.

We understand meaningful engagement in accordance with Arnstein’s “Ladder of Participation”, which broadly distinguishes between different degrees of public involvement, ranging from non-participation, to tokenism, to citizen power. In this conceptualisation, the more power citizens hold for decision-making, the better [12].

### Importance of community and stakeholder engagement in quality of care

Community and stakeholder engagement (CSE) is cited as a critical aspect of achieving universal health coverage (UHC), one of the primary outcomes of the Sustainable Development Goals [13], and for priority setting in health system decision-making and research [11]. High-quality care is an essential aspect of UHC [14–16]. Though there is no single agreed-upon definition, high-quality care is understood to be healthcare that is safe, effective, timely, equitable, efficient, and person-centred [17]. More deaths are attributable to poor-quality care than to a lack of access to health services. In 2016, it was estimated that 5 million excess deaths were due to receipt of poor-quality care (compared to 3.6 million due to non-utilisation of health care) [14]; the Latin America and Caribbean (LAC) region accounts for nearly 400,000 of these deaths [18]. CSE, therefore, presents a meaningful opportunity to improve quality of care globally, particularly where quality of care-related deaths remains high, such as in the case of Colombia.

### Colombia as a case study

Despite Colombia’s quality of care scoring lower than most other OECD countries, the country has made important progress in advancing a quality agenda and for this reason is considered to be a leader in quality of care within the LAC region [10]. Colombia is one of the few countries in the region that have a national quality of care strategic plan. As part of the movement toward achieving UHC, Colombia instituted an Obligatory System for Quality Assurance in Health Care (SOGS; Sistema Obligatorio de Garantía de Calidad de la Atención en Salud) within the framework of the reform of the National Health System in 1993 and articulated with the 2005 National Policy for the Provision of Health Services. The SOGCS put quality on the country’s healthcare system agenda and in 2011, Law 1438 was introduced, mandating that the Ministry of Health and Social Protection in Colombia define and implement a national plan for quality improvement. The first iteration of this national plan was designed in 2016 (Sistema de Salud con un propósito humano de innovación y excelencia). Following a World Bank Group external assessment of quality of care in Colombia’s health sector in 2019, the most recent iteration of the national plan was developed (Salud con calidad para todos) with an implementation timeline of 2022–2027 [9,10].

Further supporting its relevance as a case study, Colombia’s Ministry of Health has demonstrated a commitment to CSE—for example, by involving citizens in defining the health benefits package to which Colombians are entitled [19]. This commitment is also reflected in regulatory processes. As in many contexts [3], public consultation in Colombia is mandated by law; all administrative acts developed by the Ministry of Health and Social Protection must be made publicly available for comment, reviewed by a designated team, and revised in response to feedback from community members and stakeholders. Despite mechanisms for public engagement such as these, the degree to which CSE was considered in developing and implementing the national plans is not clearly understood. Therefore, this study explores how CSE has been considered in the design and implementation of Colombia’s national quality of care policies by addressing the following research questions:

1. How is community and stakeholder engagement reflected in Colombia’s National Quality of Care Strategy?
2. What approaches have been taken to engage community and stakeholders in the development and implementation of the National Quality of Care Strategy? What is their perceived effectiveness?
3. Are there opportunities to improve CSE in health policymaking in Colombia?

## Materials and methods

### Ethics statement

This research was approved by the University of Toronto’s Research Ethics Board (Protocol Reference #00044188). Formal verbal consent was obtained from all interview participants prior to interview participation. In addition, ethics permission was obtained to include the meeting minutes from consultations with key informants as part of the dataset.

### Study design

This instrumental case study [20] comprises a document analysis [21,22] followed by qualitative interviews with key informants [23] to review how CSE was reflected in and implemented through Colombia’s National Quality of Care Strategy at a national level. The instrumental case study design allowed us to use the case of Colombia to gain a broader appreciation of how CSE may be reflected in National Quality of Care Strategies. We drew on the Lavery CSE framework, which aims to clarify the components and mechanisms of CSE in practice, to guide the development of an interview guide, data analysis, and presentation of findings [11] (see Table 1).

**Table 1:** Coding frame developed from the Lavery Community and Stakeholder Engagement Framework [11].

Community & Stakeholder Engagement Components	Community & Stakeholder Engagement Subcomponents
**Foundations**	• Conceptual & ethical foundations • Enabling conditions
**Planning**	• Program goals & scope • Site selection • Forecasting relevant stakeholders & interests
**Design**	• Design constraints & decisions • Listening & deliberation • Integration with program management
**Management**	• Leadership • Human resources • Establishing & managing relationships with stakeholders • Communications
**Evaluation**	• Process • Outcomes & implications

While we considered several other engagement frameworks—including the Donabedian Model, the Institute of Medicine’s Quality Chasm Framework, and the Public Participation Framework [17,24,25] we chose to employ the Lavery Framework for this study for several key reasons. First, the Lavery Framework emphasizes engagement as the central driver of program success, particularly in public sector interventions aimed at vulnerable populations. Second, its inclusive definition of ‘community and stakeholder engagement’ encompasses all relevant parties, from health practitioners to community members, which aligns closely with both our research objectives and the perspectives of our Colombian colleagues. Third, the framework offers actionable domains, such as listening opportunities and design constraints, that facilitate direct assessment of engagement strategies, allowing for meaningful evaluation and improvement. Finally, the framework’s development is rooted in diverse international experiences, making it well-suited for application in global health contexts [8,11].

### Data collection

We conducted a document review of health policy documents, including: 1) WHO National Quality of Care Guidelines; 2) Colombian National Quality of Care Strategic documents; 3) World Bank Analyses of Quality of Care in Colombia; and 4) internal documents and presentations (Table 2). Documents were retrieved electronically through public sources and key informants. Selected excerpts from non-public documents used in the analysis are available in a public codebook included as supplementary material (S1 Appendix). In consultations aimed at generating critical background and informing the study design, valuable insights were shared with the team. As a result, ethics approval was sought to include the meeting minutes from these consultations as part of the dataset. In addition to these meeting minutes, semi-structured interviews were conducted with three individuals with involvement and/or expert knowledge pertaining to the development and implementation of the Colombian National Quality of Care Strategy to explore how the national strategy has been implemented at the national level, and how CSE has been actioned. Purposeful sampling was used to achieve variation in experience with the Strategy, and to effectively build off insights already captured in meeting minutes from earlier consultations. Potential participants were identified through the document review and the authors’ professional networks between March and June 2024. Key informants were interviewed between May and June 2024. Interviews were between 30 and 120 minutes and conducted virtually via Zoom. Spanish-language interviews were conducted, transcribed, and coded by Spanish-speaking members of the author team, who also translated relevant quotes used in this paper. The interview guide is available in both English and Spanish as supplementary material (S2 Appendix). We conducted member checking of the findings presented in this manuscript with the interviewees to further enhance the rigour and credibility of this research [26].

**Table 2:** Brief description and source of public documents included in the review.

Document	Organisation	Brief Description
Lineamiento Técnico para la Implementación del Plan Nacional de Mejoramiento de la Calidad en Salud (Technical Guidelines for the Implementation of the National Plan for Quality Improvement in Health Care)	Ministerio de Salud y Protección Social (Colombia’s Ministry of Health and Social Protection)	Colombia’s National Plan for Improving Quality of Care for the period of 2022–2027
External Assessment of Quality of Care in the Health Sector in Colombia	World Bank Group and International Finance Corporation	“A systematic assessment of the quality of health care in Colombia. The analysis covers both public and private service provision. It assesses the state of quality of care, as well as related strategies, policies, regulations and capacity to improve quality of care as a condition for the financial sustainability of the health sector. The report presents findings of this assessment as well as recommendations.[10]
Sistema de Salud con un propósito humano de innovación y excelencia (Health system with a human purpose of innovation and excellence)	Ministerio de Salud y Protección Social (Colombia’s Ministry of Health and Social Protection)	The first iteration of Colombia’s national quality of care strategy that was designed in 2016.
Law 1438 of 2011	Función Pública (Public Function)	The legal document outlining the law 1438 of 2011. The purpose of this law is to (translated from Spanish): “strengthen the General Social Security Health System through a public health service delivery model that… allows for coordinated action by the State, institutions and society to improve health and create a healthy environment that provides higher quality, inclusive and equitable services, where the focus and objective of all efforts are the residents of the country.” [27]
Participación social en salud Resolution 2063 of 2017	Ministerio de Salud y Protección Social (Colombia’s Ministry of Health and Social Protection)	Webpage. The stated objective of the site is to (translated from Spanish): “transform the relationship between citizens and the Ministry of Health and Social Protection, helping them participate in the formulation of programs, plans, projects and public policies in the health and social protection sector. All citizens can participate regardless of their gender, race or social status; Likewise, organizations and civil society can also participate.” [28] It is on this webpage that the national strategy (2022–2027) was posted for public feedback.
Manual Operativo del Programa: Mejorar el Acceso Efectivo de la Población Vulnerable a Servicios de Salud y Aumentar la Resiliencia del Sistema de Salud, 2024–2026 (Program Operative Manual: Improve Effective Access of Vulnerable Populations to Health Services and Increase Health System Resilience)	World Bank Group and Ministerio de Salud y Protección Social (Colombia’s Ministry of Health and Social Protection)	The manual that outlines the rules and regulations tied to recently funded programming under Colombia’s Ministry of Health and Social Protection.

### Data analysis

We undertook an iterative approach to document analysis that included an initial rapid scan of documents to identify and categorise data related to our research aims, followed by a close coding of the text in which we used a coding frame developed from Lavery’s CSE Framework (Table 1) [11,21]. An initial review of the documents was used to inform the interview guide. Once data collection had concluded, both sets of data (documents and interview transcripts) were coded using directed content analysis as well as open coding techniques to capture emergent themes. For the directed content analysis, constructs from the Lavery CSE Framework were used as sensitising concepts in which data sources were coded for each component and subcomponent of the framework [11]. To develop a holistic understanding of the research objectives, codes for each component were then analysed jointly across all datasets (data triangulation) [20].

## Results

The following sub-sections outline our findings, organised by Lavery’s five community and stakeholder engagement components: foundations, planning, design, management, and evaluation [11]. A summary of the research findings is available as supplementary material (S1 Fig).

### Foundations

The Foundations section of the Lavery framework focuses on three engagement components: 1) Conceptual Foundations, 2) Ethical Foundations, and 3) Enabling Conditions. The explicit intention to engage the community and relevant stakeholders, and the high valuation of their participation, is clear throughout Colombia’s 2022–2027 National Plan to Improve Quality of Care (Plan Nacional de Mejoramiento de la Calidad de Salud, PNMCS). The revisions of the updated 5-year national plan were driven by two categories of problems: 1) problems that persisted from the 2016–2021 quality of care plan (e.g., difficulty in users accessing information and a “legitimacy and trust crisis” (PNMCS, p. 10)), and 2) emerging problems in the Colombian healthcare system.

The development of the 2022–2027 Strategy itself was initially informed by OECD indicators, the Triple Aim [25] and the WHO Handbook for National Quality Policy and Strategy (NQPS), before being shared with stakeholders (described in detail in the Planning section of this article). The NQPS and OECD guidelines highlight public and citizen engagement as key principles for high-quality health systems, which influenced the steadfast inclusion of CSE throughout the Strategy. An external assessment of quality of care in the health sector in Colombia likewise stated the importance of patient and citizen involvement to “lead the revolution that is required to ensure that a high-quality health system in Colombia delivers quality to all Colombians” ([16] pg. 59). The 5-year Strategy clearly defines patient/ family/ community participation as:

“Focus on care that deliberately takes the point of view of individuals, families and communities and considers them as participants and beneficiaries of health systems.” (PNMCS pg. 26, translated from Spanish)

Incorporation of patient engagement was also noted by key informants and in the PNMCS as driven by Colombia’s 2011 Law 1438, which states that “health services must address the patient’s conditions in accordance with scientific evidence, provided in a comprehensive, safe and timely manner, through humanised care” (PNMCS pg. 2, translated). The humanisation of health is named as a fundamental pillar of healthcare services, and one of the eight “lines” of focus in executing the Strategy on healthcare that is focused on centring health services around people, family, and community [9].

The World Bank assessment of quality of care in Colombia recommended engaging patients and citizens by providing them with localised information on quality, establishing benchmark quality indicators for providers and insurance companies and making these transparent to the public (e.g., through a provider star rating or ranking system), strengthening patient participation in planning processes, and developing accountability mechanisms [10]. The Strategy explicitly recognises the importance of citizen participation that is inclusive of all Colombians, stating that citizen participation is “where citizens, organisations, and civil society participate in the formulation of programs, plans, projects, and public policies in the health and social protection sector, regardless of their gender, race, or social status” (PNMCS, pg. 15).

The written foundations for CSE for quality of care in Colombia are substantial and clear. However, as one key informant stated when reflecting on steps required beyond written commitment, “in order to implement quality, it needs [to be ingrained in the health system] culture,” a shift they felt had not yet been achieved in the Colombian health system.

### Planning

Planning for CSE entails: 1) defining the program goals and scope, 2) clarifying site selection, and 3) forecasting relevant stakeholders and interests [11]. As per requirements set by the Colombian government, National Resolutions need to be publicly shared through government websites with the general public and interested stakeholders for review and comment. This opens an avenue for engagement of the public in national programs, while also creating accountability on behalf of the administration to respond to comments and revise resolutions, if necessary.

The Colombian Ministry of Health invites communication on new policy documents and guidelines, including the PNMCS, in two waves. First, documents are shared with key internal stakeholders within the Ministry of Health and other governmental ministries. Internal stakeholders are invited to review new documents and propose changes; they are then informed about whether their edits were accepted and why. Following the internal review, the Ministry must post the document for public review and comments for a period of 15 business days. As one key informant describes:

“For a document to come out of the ministry, there must be a resolution, an administrative act, something that makes it formal. The document is delivered with a justification written by the public servant on why the document exists, when it was published, what feedback was received and what was done with the feedback? [The public servant] has to respond to whoever provides feedback, to anyone. The minimum response is to say thank you for the interest and explain whether the feedback is accepted or not.” Key Informant 1, interview, translated from Spanish)

For the internal review of the PNMCS, six live sessions were held with a maximum of 30 participants each to provide an overview of the document and allow for a question-and-answer period. Following the internal review of the PNMCS, key stakeholders were actively engaged to comment on the document; this included individuals at the Pan American Health Organization, healthcare representatives and providers, insurance agencies, and user associations.

The public and other external stakeholders were engaged passively through the public posting of the document. Additional passive engagement efforts included public sharing of data on the official website of the Ministry of Health and Social Protection, which included descriptions of ongoing projects, reports, and findings, as well as individuals and agencies that are involved. However, there was limited information on the extent of public awareness regarding these posted resources; one key informant recalled that only about four contributions were received from the public, and all were focused on clarifying certain wording within the Strategy document. Here, we describe public engagement as passive because there was a lack of promotion efforts when the policy documents were posted online for feedback.

Key informants had differing beliefs about the extent to which patients and citizens could contribute to the planning of the Strategy. One stated that the definition of ‘community’ in this context was very broad, including both the public as well as hospital and healthcare staff and secretaries. In addition, they mentioned that discussions surrounding quality of care could be technical, and difficult to discuss with individuals without familiarity with the healthcare context. Yet, key informants maintained that the general public is an essential stakeholder in planning for quality of care in Colombia:

“Who is impacted by what we define as quality? Everyone, to whom it is reported and to whom it is applied, which is the user and their family and the broader community…So the challenge was how to co-construct the plan, how to make it understandable, useful, and practical for everyone.” (Key Informant 1, translated from Spanish)

Key informants emphasised a need for future innovation in engagement strategies to better include the perspectives of both policymakers and care providers, as well as members of the public of varying socioeconomic status.

### Design

The Lavery framework sub-section on Design focuses on three components of CSE strategies: 1) Design Constraints and Decisions, 2) Listening and Deliberation, and 3) Integration with Program Management [11].

One of the strengths of Colombia’s National Quality of Care Strategy lies in the provision of eight clear lines of action for the healthcare system. In addition to identifying areas of priority, the Strategy provides practical engagement tools that can be used during implementation. These tools are provided in a tiered format, with ‘basic, optimum and excellent’ levels, accounting for differences in available resources that exist between regions.

In the Strategy, Colombia’s Ministry of Health and Social Protection (Ministerio de Salud y Protección Social) identifies points of concern with the current quality of care plan, some of which were also a challenge with the 2016–2021 plan. In developing and implementing the CSE strategy, the Ministry recognises challenges in having the general public access information necessary to facilitate engagement, and the insufficient recognition of the different health needs of specific populations, including women, racialised people, and lesbian, gay, bisexual, transgender, queer, and/or intersex (LGBTQI+) people. These concerns illustrate a lack of built-in opportunities for listening to patients and citizens, and in particular those who belong to equity-deserving groups. Effective listening strategies are key to CSE [11]. In its report, the Ministry of Health and Social Protection indicates an intention to generate “spaces for social participation to provide inputs in the next update of the National Quality Improvement Plan of Colombia” (PNMCS, p. 31), however, specific detail is not provided as to how this will be executed.

Key informants shared challenges in creating spaces for public engagement. For example, virtual engagement was the only format possible, as the plan was developed in the early stages of the COVID-19 pandemic when public health restrictions were in full force. National scope was noted as another challenge; one key informant described the vast number of stakeholders that could be involved in quality discussions, noting that Colombia has over 11,000 hospitals and a wide array of healthcare staff and authorities to consider in addition to the general public. Furthermore, key informants shared the challenge and need to recognise and consider the differences in populations across Colombia’s different regions. Without consideration of these differences, CSE may unintentionally exclude specific groups from consideration in developing and implementing quality of care plans, resulting in a sorely lacking perspective from women, Indigenous people, Afro-descendants, LGBTQI+ people, and others. The government of Colombia is currently taking steps to be more inclusive of structurally marginalized voices, particularly by engaging with Indigenous communities, such as through the development of the Sistema Indígena de Salud Propio e Intercultural (Indigenous and Intercultural Health System) [29]. One key informant describes efforts to engage Indigenous health practices:

“Indigenous communities have their own health practices… and Colombia is trying to integrate these practices within the main health system. So many channels have been [recently] opened with these groups to learn more about their own health systems or their own health practices and how these could be integrated [with Colombia’s health system].” (Key Informant 3, translated from Spanish)

### Management

Management of CSE encompasses aspects relating to leadership, human resources, establishing and maintaining relationships with stakeholders, communications, and establishing a presence within communities [11]. Key informants confirmed that Colombia employed a combination of active and passive engagement strategies in developing and implementing the Strategy, but that it was easier to actively engage with known stakeholders such as healthcare practitioners or ministry officials.

“Another problem that we identified is that quality is focused on institutions, on insurers, but not on people. The issue of a people-centred approach to care does not exist.” (Key Informant 1, translated from Spanish)

For example, since there is high turnover of staff in Colombia’s health care sector, the Ministry of Health requires territorial health directorates to periodically visit healthcare centers to verify the quality of care provided, which requires a constant effort to ensure providers are up to date on guidelines relating to quality of care. Citizens are more apt to be engaged through passive approaches or identified “capsules” of populations (defined by actors within their territories) and their representatives. This was made clear as only user associations were actively engaged in the development of the strategy, as described earlier in the sub-section Planning.

Key informants noted that there is very little financial or material support for CSE, and that engagement with the public happens only because there are leaders who champion this work within the quality department of the Ministry of Health and Social Protection. Government bureaucracy was noted to create a barrier to CSE:

“No, there is no support. In fact, sometimes there’s barriers rather than support… We had difficulty getting the Quality Office [set up] to allow people to spend their time on this… There were more barriers than incentives or support.” (Key Informant 3, translated from Spanish)

Historically in Colombia, quality of care policies have used punitive measures to reinforce quality of care standards among healthcare practitioners, rather than support excellent results; one key informant noted that this kind of “punitive approach to quality of care… goes against quality developments” and can hinder engagement opportunities. In a welcomed change, the 2022–2027 PNMCS instead outlines plans to give awards and publicise health sector agents that have excellent results, even suggesting provision of prizes for healthcare transparency.

### Evaluation

The Lavery framework sub-section on evaluation outlines both process evaluation and outcomes and implications [11]. Our assessment identified that minimal process or outcome evaluation of the CSE processes was undertaken or was otherwise not available.

The World Bank assessment of quality of care in Colombia found that the earlier strategy lacked clarity in outlining “targets, plans, activities, roles and responsibilities, and accountability for results,” and also noted that publicly available health data is often released without sufficient context, making it difficult to interpret and translate into clear action items ([10] pg. 10). The Strategy, developed following this external assessment, has made notable improvements in articulating its plan, targets, activities, and roles and responsibilities. However, the effectiveness of implemented CSE strategies remains unclear. Much of community engagement has been passive in nature, limited primarily to the public posting of the Strategy document and contextual project data, reports, and updates. While these materials offer more context than what was previously provided by the Ministry, the absence of built-in mechanisms to evaluate community engagement makes it difficult to determine whether they offered sufficient context or the type of information needed to support meaningful public participation. As such, questions remain about who (beyond professional stakeholders) is being reached through these approaches, how accessible or actionable the information is, and whether it contributes to meaningful change.

Consistent with past engagement efforts, key informants noted that the evaluation tools outlined in the Strategy are similarly structured to prioritize engagement with health system professionals over direct involvement of community members (i.e., patients and citizens):

“What we did with the plan was provide tools and stagger the tools to indicate, at the basic level what is expected, at the optimum level what is expected and at the excellent level... so that in the self-assessment each provider actor, insurer, territorial departments can locate themselves as to where they are: basic, optimal, or excellent [as their starting point]. Tools are provided based on international and national evidence on successful evidence [to help them move forward from that point].” (Key Informant 1, translated from Spanish)

## Discussion

This instrumental case study of community and stakeholder engagement (CSE) in the development and implementation of the National Quality of Care Strategy in Colombia illustrates both the clear and multi-faceted commitment of the Colombian Ministry of Health to engaging the public in the development of policies and the persistent challenges in translating that commitment into meaningful and inclusive action. While CSE is explicitly valued within the policy framework, our findings underscore that policy commitments alone do not guarantee implementation, particularly in engaging community (i.e., patients and citizens). The realization of meaningful engagement requires sustained political will, dedicated resources, and robust systems for evaluation and feedback.

Although the Strategy demonstrates a strong and detailed written commitment to CSE in the development of quality-of-care policies, implementation has not always translated into action in a coherent way. Engagement approaches have been underfunded and difficult to track, creating barriers to the meaningful engagement of patients and citizens in public policy development. One such example is the predominance of passive engagement approaches, such as posting the strategy online for public comment. Without effective awareness strategies, it is unsurprising that these methods limit engagement to professional groups and employees within the public sector and healthcare, to the exclusion of patients and citizens who would not be aware of this feedback opportunity. This has particular implications that contribute to the exclusion of structurally marginalised populations from the policy process [30]. Research on public participation has consistently shown that engagement initiatives often fail to reach marginalised populations, such as low-income individuals, individuals with lower levels of education, and racial and ethnic minorities [31]. This has prompted researchers to call for targeted frameworks and strategies to engage marginalised groups, to avoid reinforcing existing inequities by amplifying the voices of the already-empowered while sidelining those most affected by gaps in healthcare [32,33]. For engagement efforts to be meaningful, power must be shared with frontline public servants responsible for engagement activities and the citizens and communities with whom they engage [34]. Steps currently taken by the Colombian government to actively engage user associations and to elevate Indigenous voices in health policy and decision-making are important first steps, but broader inclusion efforts remain needed.

Despite the many strengths in the ‘foundations’ of support for CSE within Colombia’s quality-of-care agenda, our findings reflect a broader pattern in Colombian health policymaking. As key informants noted, ever-changing government officials and the vacillation of political support for quality healthcare is a challenge in maintaining momentum for the PNMCS and related interventions. These challenges are emblematic of broader governance issues described in Colombia’s health sector, where decentralization and a highly fragmented polity often hinder effective implementation of national strategies [35,36]. Limited resources and financial support for engagement activities and their evaluation further weakens efforts to foster meaningful and inclusive CSE, particularly among “seldom heard” or marginalised populations [30]. Our findings, therefore, reveal a critical yet typical disconnect between the Strategy and ongoing engagement, highlighting the need for more resilient institutional mechanisms that can buffer national strategies and CSE processes from fluctuating political commitment.

Our analysis demonstrates a clear need to consider barriers to the implementation of CSE strategies (e.g., change in political leadership, lack of dedicated CSE teams, and limited resources) at the design stage. While writing CSE activities into the design of public policies is a positive start, intention alone is insufficient. CSE design must involve planning around the availability (or lack thereof) of necessary resources to execute the proposed CSE activities. Evaluation of which CSE activities are conducted, what worked, and why is another key consideration, and can be guided by implementation science frameworks or a CSE-specific framework. As was acknowledged by key informants, limited engagement with structurally marginalised populations points to a need to develop alternative or targeted engagement strategies that better reach these populations. In the case of Colombia, this could include targeted advertisement of the public posting of the Strategy and targeted engagement activities with these communities.

Furthermore, Colombia’s national healthcare satisfaction survey (Encuesta Nacional de Satisfacción de los Usuarios Frente a los Servicias de las EPS)—which includes disaggregated data by region, sex, socioeconomic status, rurality, and insurance coverage (i.e., contributory or subsidized)—presents a valuable opportunity to identify priority regions or marginalised groups for quality of care improvement. This data can be used to more effectively allocate limited resources for targeted CSE strategies. Although data systems and technical capacity at the local level are limited, as noted in national policy analyses [37], rich national-level data such as this can play an important role in supporting more equitable and evidence-based decision-making around CSE by narrowing the pool for targeted engagement.

## Strengths and limitations

This study has several notable strengths. First, the use of an instrumental case study design incorporating multiple data sources (policy documents, internal reports, interviews and meeting minutes) enabled methodological triangulation and added depth and credibility to our findings. Second, we applied the Lavery framework, an established and internationally recognized model for CSE, which provided conceptual clarity and a structured coding approach to guide our analysis. Third, member checking with interview participants further enhanced the trustworthiness of our interpretations. Lastly, this study addresses a critical gap in the literature regarding how CSE is operationalized in national policy development within low-and middle-income countries, offering timely insights relevant to global health policy and for Colombia and comparable jurisdictions in country efforts toward achieving universal health coverage.

However, this study is not without its limitations. Our analysis was limited to the national level, when many of the effects of CSE are experienced at sub-national and community levels. Further, our analysis misses key perspectives, for example from healthcare administrators, clinicians, and members of the public themselves. Finally, the timing of our analysis does limit the extent to which we were able to assess CSE strategies during the implementation stage of the strategy, as implementation is still ongoing. Future research may focus on a sub-national context and offer a comparative evaluation at a later stage in the Strategy’s implementation.

## Conclusion

Overall, Colombia’s National Quality of Care Strategy provides a strong example for other countries seeking to develop a national plan rooted in community and stakeholder-identified priorities. However, inconsistent political and financial support has hindered the plan from reaching its full potential. Future research evaluating the feasibility of different CSE strategies in designing and implementing health policies is needed in Colombia.

## Supporting information

S1 Appendix Codebook. (PDF)

S2 Appendix Semi-structured Interview Guide (Available in English and Spanish). (PDF)

S1 Fig Summary of Research Findings (Images designed by Freepik). (EPS)
