# Supplementary material for: Leveraging public engagement to improve healthcare quality: The role of community and stakeholder engagement in Colombia’s National Quality of Care Strategy
Source: PLOS Glob Public Health. 2025 Nov 4;5(11):e0005333. doi: 10.1371/journal.pgph.0005333 (PMC12585033; doi:10.1371/journal.pgph.0005333)
Supplement: S1 Appendix — (PDF) [file pgph.0005333.s001.pdf]

CSE Component	Example Excerpts
Foundations	
Conceptual Foundations	There are many principles guiding the PNMCS. People-centered care is listed as the first principle.<br>• People-centred care is defined as a: 'focus on care that deliberately takes the point of view of individuals, families and communities and considers them as participants and beneficiaries of health systems.' ((1) pg. 26, translated from Spanish)<br>Other relevant principles include Dignity, Equality, Humanization in Health, Transparency and Citizen participation.<br>• Citizen participation is defined as: 'where citizens, organisations, and civil society participate in the formulation of programs, plans, projects, and public policies in the health and social protection sector, regardless of their gender, race, or social status' ((1), pg. 15, translated from Spanish)<br><br>The NQPS and OECD guidelines and World Bank evaluation, on which the Strategy is based, highlight public and citizen engagement as key principles for high-quality health systems, which influenced the steadfast inclusion of CSE throughout the Strategy.<br>• The World Bank evaluation recommends patients and citizens all be fully engaged and 'lead the revolution that is required to ensure that a high-quality health system in Colombia delivers quality to all Colombians' ((2) pg. 59)<br>• The World Bank evaluation defines patient-centeredness as 'Providing care that is respectful of and responsive to individual patient preferences, needs, and values and ensuring that patient values guide all clinical decisions' ((2), pg 52)<br><br>Key informants speak to the basis for and value of engagement strategies:<br>• It was important for government to find a framework of quality, and they decided to use the Triple Aim Framework (Meeting minutes)<br>• In developing the strategy, a key point emphasized by government officials was the need to achieve just access to health services. (Meeting minutes)
Ethical Foundations	The Strategy emphasizes public engagement, focusing care on people, families, and communities:<br>• 'The guarantee that people have informed participation in decisions related to their health' is mentioned when describing the pillars of the national plan ((1), pg 8, translated from Spanish)<br>• The "Humanization of Health" is listed a fundamental pillar for healthcare and services ((1), pg 9, translated from Spanish)<br>• The Strategy also highlights the need to 'systematically and incrementally innovate and change over time', incorporating the experiences of agents ((1), pg 12)<br><br>In speaking about the Strategy, Key Informants note:<br>• 'The first quality problem found in Colombia is that there are many unknown quality standards, and different interpretations. Another problem that we identified is that quality is focused on institutions, on insurers, but not on people. The issue of people-centred approach to care does not exist.' (Key Informant 1, translated from Spanish)<br>• 'Excellence in service…we do not want to stay in the basics, but instead strive for excellence. Here, as you know, there is an accreditation model, but the invitation [for healthcare professionals with this Strategy] is that whatever leads [them] toward excellence [in quality of care] is useful.' (Key Informant 1, translated from Spanish)
Enabling Conditions	• 'There is a mandatory system that we already mentioned with four components: authorization, accreditation, information system, and auditing. And there is Law 1438, which is the one that says that mandates that the National Quality Improvement Plan centres patient care.' (Key Informant 1, translated from Spanish)

	• Colombia's 2011 Law 1438: 'health services must address the patient's conditions in accordance with scientific evidence, provided in a comprehensive, safe and timely manner, through humanised care' ((1) pg. 2, translated from Spanish)<br><br>Colombia's legislation (Resolution 2063 of 2017) mandates public posting of national strategies:<br>• The stated objective of the resolution is to: 'transform the relationship between citizens and the Ministry of Health and Social Protection, helping them participate in the formulation of programs, plans, projects and public policies in the health and social protection sector. All citizens can participate regardless of their gender, race or social status; Likewise, organizations and civil society can also participate.' ((3), translated from Spanish) It is on this webpage that the national strategy (2022-2027) was posted for public feedback.<br>• 'The moment [the National Quality of Care Strategy] leaves the national level, everyone can access it, because... all national strategies must be made public, and the Ministry has the obligation to publish it on its page.' (Key Informant 1, translated from Spanish)
Planning	
Defining the program goals and scope	Colombia's Strategy establishes eight lines of action (including primary care, patient safety, people-centered care, and search for excellence) and introduces practical tools and self-assessment benchmarks at basic, optimal, and excellent levels to guide continuous improvement.<br>• 'What we did with this new plan is to give some lines of action, there are eight... We focus on eight lines and the big difference in this plan is that in the second part we give [healthcare practitioners] practical tools. We say how to [achieve better quality of care delivery] and offer different starting points. Colombia is a country of diverse regions, a lot of progress at the central level, in Bogotá, and a lot of setbacks in other parts, like in remote areas. What we did with the plan was provide tools and stagger the tools to indicate, at the basic level what is expected, at the optimum level what is expected and at the excellent level... so that in the self-assessment each provider actor, insurer, territorial departments can locate themselves as to where they are: basic, optimal, or excellent [as their starting point]. Tools are provided based on international and national evidence on successful evidence [to help them move forward from that point].' (Key Informant 1, translated from Spanish)
Forecasting relevant stakeholders and interests	• The central government is in a unique position to identify challenges and needs in the region. Relevant stakeholders identified by the government include local authorities, healthcare staff and the general public. However the magnitude of relevant stakeholders for engagement is noted as a challenge: 'we have more than 11,000 hospitals in Colombia, big and small. So that's a lot of people in which you can engage in conversation. And then you have the general public.' (Meeting minutes)<br>• 'Who is impacted by what we define as quality? Everyone, to whom it is reported and to whom it is applied, which is the user and their family and the broader community…So the challenge was how to co-construct. How do we make it digestible, useful, and practical for everyone?' (Key Informant 1, translated from Spanish)
Design	
Design Constraints and Decisions	The Strategy highlights ongoing challenges in user access to information and recognition of diverse health needs, especially among equity-deserving groups, reflecting a lack of built-in opportunities for listening to service users:<br>• 'The focus of continual improvement is focused on system agents rather than people' / - 'Difficulty in users accessing information'/ 'Legitimacy and trust crisis' / 'Lack of patient and user participation in decisions related to their health, and little recognition of the different needs of ethnic and racialized peoples' (footnote says, including women, LGBTQI+) ((1), pg 10, translated from Spanish)

	• 'Today, there is a growing focus on quality... It is mandatory to have a national plan aimed at improving quality across the country. As [Name of Key Informant] would have explained, this plan acts as an "umbrella" that encompasses the broader quality ecosystem. It aims to make the movement toward achieving quality of care more dynamic. When regulations feel too mandatory, they often generate resistance and lead to concealment, manipulation, or even falsification of data, practices that ultimately lead to poor quality of care.' (Key Informant 1, translated from Spanish)
Listening and Deliberation	Colombia is working to include marginalized voices, especially Indigenous communities, through initiatives like the Sistema Indígena de Salud Propio e Intercultural.<br><br>• 'Indigenous communities have their own health practices… and Colombia is trying to integrate these practices within the main health system. So many channels have been [recently] opened with these groups to learn more about their own health systems or their own health practices and how these could be integrated [with Colombia's health system].' (Key Informant 3, translated from Spanish)<br><br>The Ministry of Health's report notes the intention to create "spaces for social participation" in updating the National Quality Improvement Plan but lacks specific details:<br>• 'Generation of spaces for social participation to provide inputs in the next update of the National Quality Improvement Plan of Colombia.' ((1), pg 31)<br><br>• 'The challenge was figuring out how to co-construct the plan, how to make it understandable, useful, and practical for everyone. So, we started by revisiting an evaluation that had been done on the previous plan. That evaluation revealed that many people weren't even aware of the plan... So we carried out a review of a lot of information sources: data from the quality information system, interviews with institutions, and conversations with individuals who had successfully improved quality, particularly those from accredited organizations.' (Key Informant 1, translated from Spanish)<br><br>• 'Q: Once again, the people who were in the interviews, it was the Ministry of Health and then?<br>Key Informant 1: The Ministry of Health, insurers, and providers were all involved. Remember, there are four types of providers here, which are hospitals, clinics, public hospitals, and independent professionals. It's especially important to include independent professionals, because they often feel disconnected from quality initiatives. They tend to think quality is only relevant for large clinics or hospitals. Ambulance services were also included, because they are required to meet quality standards as well.<br>We also reached out to places that don't provide direct health services but still have medical offices for the public, like with universities, airports, and libraries. These organizations must also ensure quality in their healthcare services, no matter how limited.<br>This entire process took place in a virtual environment... we were in the pandemic. We have them a document and then they could give their opinions, like "this applies, this not so much, this I do not understand". And then adjustments were made, and together we developed this guideline for these stakeholders to use.' (Translated from Spanish)
Integration with Program Management	Efforts were made to mobilize stakeholders as active allies in quality improvement initiatives:<br>• 'Which of the eight lines did you prioritize? Which one?<br>– "I'm working in primary care, so I chose patient safety."<br>– "I chose excellence."<br>• Ah, so they responded. That allows us to see which lines have had the most impact. Then we asked: Are you using the products provided here, or are you doing something different? Some answered, "I'm using the capsules guideline," or "I'm

	using the empowered guideline.” Others said, 'I’m not using any of those, I developed my own approach.’ All of this was part of the initial phase of implementation, aimed at identifying where quality efforts are headed and how the different lines are being adopted.’ (Key Informant 1, translated from Spanish)
Management	
Leadership	Key informants noted minimal financial support for CSE, with public engagement relying largely on dedicated leaders within the Ministry’s quality department:<br>• ‘No, there is no support. In fact, sometimes there's barriers rather than support… We had difficulty getting the Quality Office [set up] to allow people to spend their time on this… There were more barriers than incentives or support.’ (Key Informant 3, translated from Spanish)<br>• The PNMCS outlines plans to give awards and publicize health sector agents that have excellent results when carrying out the Strategy. ((1), pg. 32, translated from Spanish)
Human resources	The Ministry of Health’s dedicated quality department drives efforts, but high staff turnover in Colombia’s healthcare sector challenges consistency.<br><br>The Quality Plan was developed through a collaborative validation process, engaging health workers and institutions across different levels of care (low, intermediate, and high complexity), as well as insurers.<br><br>• ‘We generated a document, a tool in Google Forms where we asked them: Do you consider the document useful? Yes or no. Is it relevant or is it important? And, questions along the lines of, do you consider the priority lines that we selected to be relevant? Are they relevant, are they useful? And with the responses, we did statistical analysis, basically [trying to understand]: what should we remove, what should we leave, what should we strengthen. More and more we were expanding the group [we engaged]. With the consensus of all areas of the ministry, we invited different actors [to participate]. Since there are databases, we would try to have representation from [health system actors] with low complexity, those furthest away, those at intermediate levels and those with high levels of complexity... And then if they did not go, for example, to the virtual meetings, we sent them the document with the explanation of why it was important for them to participate in the validation, asked that they read it and to tell us if they found it important, relevant and useful... The pleasant surprise is that it was always between 4.5 and 5...In other words, it was very well received. With that... we took the observations into account again. There are people, for example, from Primary Health Care, who made very nice contributions. But we also took into account that the scope of the plan is not absolute, because many would like to "put this, put this, put that"... “put something about migrants”... and we *are* talking about equity. [Marginalized groups] do all come into consideration... that is what, for example, the Ten-Year Public Health Plan or other plans are for. What we do say, frankly, is that this plan is in line with all those others that are working simultaneously. We cleaned the document, we reviewed it again, we included what we considered pertinent with scientific evidence, and we published it on the Ministry's website so that the entire population could have an opinion.’ (Key Informant 1, translated from Spanish)
Establishing and maintaining relationships with stakeholders	Key informants noted that it was easier to actively engage with known stakeholders (e.g., clinicians, ministry officials):<br>• ‘Another problem that we identified is that quality is focused on institutions, on insurers, but not on people. The issue of a people-centred approach to care does not exist.’ (Key Informant 1, translated from Spanish)<br>• Due to frequent staff turnover in local health authorities, the Ministry must continuously re-engage and educate regional personnel about quality, making ongoing outreach and communication essential for sustained engagement. (Meeting minutes)

Communications	• Six live sessions (max 30 participants each) were held to present the document and gather feedback, followed by active stakeholder engagement after the internal review of the PNMCS. (Meeting minutes)<br><br>The Ministry of Health's "Participación Social en Salud" website seeks to transform citizen–government relations by opening participation in policy development and providing transparent access to detailed project documents, however:<br>• Translating the technical language of quality of care into clear, understandable terms is a major challenge in way of involving patients and citizens in quality of care discussions. Efforts to address this include using media and simplified tools, such as the 'instruments used to measure the quality of care, to select the APS or the provider.' this is the first instrument the Colombian government made to engage the public in these discussions, but generally, engagement with the public is limited. (Meeting minutes)
Establishing a presence within communities	• Territorial health directorates must regularly visit healthcare centers to verify care quality and ensure providers stay updated on guidelines. (Meeting Minutes)
Evaluation	
Process evaluation	• The government leverages national surveys to collect data on quality of care. (Meeting Minutes)<br>○ High satisfaction (73% in 2017), higher in the subsidized (77%) vs. contributory (67%) regimes; main concerns include long wait times, slow insurance approvals, and limited measures of person-centeredness. Public reporting exists but is often outdated, overly technical, and hard for consumers to use effectively. ((1, 2, 4))<br>• 'The Communications Office at the Ministry has a defined process for document review. Once a document is ready, it's shared with two public servants who wait for feedback to come in using a specific tool. This tool asks: What page? What section? What is your observation? What change do you propose? Once all of the feedback is collected, the technical team.. usually the original three authors.. reviews the relevance of each comment and revises the document accordingly. For a document to come out of the ministry, there must be a resolution, an administrative act, something that makes it formal. The document is delivered with a justification written by the public servant on why the document exists, when it was published, what feedback was received and what was done with the feedback? [The public servant] has to respond to whoever provides feedback, to anyone. The minimum response is to say thank you for the interest and explain whether the feedback is accepted or not.' (Key Informant 1, translated from Spanish)

References

1. Ministerio de Salud y Protección Social C. LINEAMIENTO TÉCNICO PARA LA IMPLEMENTACIÓN DEL PLAN NACIONAL DE MEJORAMIENTO DE LA CALIDAD EN SALUD 2022 - 2027. 2021 2021.
2. World B. External assessment of quality of care in the health sector in Colombia. World Bank. 2019.
3. Ministerio de Salud Pública y Protección S. Transparencia y acceso a la información pública.
4. Social; MdSyP. Encuesta Nacional de Evaluacion de los Servicios de las EPS Colombia2017 [Available from: https://www.minsalud.gov.co/sites/rid/Lists/BibliotecaDigital/RIDE/DE/CA/Informe-encuesta-satisfaccion-eps-2017.pdf.
