# Supplementary material for: Leveraging public engagement to improve healthcare quality: The role of community and stakeholder engagement in Colombia’s National Quality of Care Strategy
Source: PLOS Glob Public Health. 2025 Nov 4;5(11):e0005333. doi: 10.1371/journal.pgph.0005333 (PMC12585033; doi:10.1371/journal.pgph.0005333)
Supplement: S2 Appendix — (PDF) [file pgph.0005333.s002.pdf]

Interview Guide in English

Opening Questions

1. What is your current position? [Tell us about the work that you do.]
    a. What was your position during the development of the National Quality of Care Plan?
    b. What role did you play in its development?

Developing the National QoC Plan

2. In what ways was citizen/stakeholder/public engagement a priority in developing the National Quality of Care Plan?
    a. Who was/was not prioritized for public engagement? Why / why not?
        i. Funders, researchers, stakeholders

3. Could you tell me about the citizen/stakeholder/public engagement strategies used to inform the development of the National Quality of Care Plan?
    a. Which stakeholder groups did they focus on including? (e.g., clinic administration, healthcare practitioners, patients)?
        i. Were any key stakeholder groups left out? If yes, who?
        ii. Given Colombia's regional diversity (in terms of health needs/demographics), were there tailored strategies according to regional differences?
        iii. Were there efforts to capture perspectives from structurally marginalized (vulnerable/marginalized) populations? If not, why?
    b. What was the focus of the engagement strategy?
        i. What was the scope of the engagement?
    c. Were these successful? Why or why not?
        i. Approximately how many people/respondents were engaged through the discussed strategy?

4. What form(s) of support did the team receive for the engagement strategy/ies?
    a. What form(s) of support would have enabled improved engagement?
        i. The degree to which engagement was prioritized/valued
        ii. Whether there was(n't) a dedicated team focused on engagement
        iii. Associated costs
        iv. Were there honoraria/ other incentives to participate in the engagement strategies?

5. Could you tell me (more) about the team responsible for engagement?
    a. What types of leadership and skills are present/missing?

6. Once feedback was received from the citizen/stakeholder/public, how was this then implemented/considered in the development of the National Quality of Care Plan?

Looking Ahead

7. Could you please share some opportunities to improve engagement going forward?

8. For interests that were not identified/ addressed (missing citizen/stakeholder perspectives), how might these groups be better engaged and their interests better addressed in the future?

9. What difference was made by the engagement of the public and community in the development of Colombia's national quality of care strategy?

10. In your opinion, in what ways was the overall engagement effective?
    a. Whose (citizens/stakeholders/public) interests have been identified and acknowledged?

Evolution of Colombia's Quality of Care Strategy

11. In comparing the National QoC plans (2014 versus 2021), we see that there has been more reference to engagement in the current plan. Could you tell me a bit about how this change came about?
    a. Who was involved in developing both strategies? How did the team(s) change?
    b. Were there criticisms of (the lack of) engagement strategies in the 2014 plan?
        i. If yes, how were(n't) these addressed in the updated plan?

Closing Questions

12. What does meaningful public and community engagement in health systems change look like to you?

13. What do you think other contexts/countries could learn from Colombia's efforts?

14. Do you know of other contexts/countries with strong examples of citizen/stakeholder/public engagement in the development of quality of care strategies?

Guía de entrevista en Español

Preguntas iniciales

1. ¿Cuál es tu posición actual? [Cuéntenos sobre el trabajo que realiza.]
    a. ¿Cuál fue su posición durante el desarrollo del Plan Nacional de Calidad de Atención?
    b. ¿Qué papel tuviste en su desarrollo?

Desarrollo del Plan Nacional de Calidad de la Atención

1. ¿De qué manera la participación de los ciudadanos/partes interesadas/público fue una prioridad en el desarrollo del Plan Nacional de Calidad de la Atención?
    a. ¿A quién se le dio o no prioridad para la participación pública? ¿Por qué / por qué no?
        i. Financiadores, investigadores, partes interesadas

2. ¿Podría contarme sobre las estrategias de participación ciudadana/actor/público utilizadas para informar el desarrollo del Plan Nacional de Calidad de la Atención?
    a. ¿En qué grupos de partes interesadas se centraron en incluir? (¿por ejemplo, administración de la clínica, profesionales de la salud, pacientes?)
        i. ¿Quedó fuera algún grupo de partes interesadas clave? Si es así, ¿quién?
        ii. Con la diversidad regional de Colombia (en términos de necesidades de salud/demografía), ¿existieron estrategias adaptadas a las diferencias regionales?
        iii. ¿Hubo esfuerzos para captar las perspectivas de poblaciones estructuralmente marginadas (vulnerables/marginadas)? Si no, ¿por qué?
    b. ¿Cuál fue el enfoque de la estrategia de participación?
        i. ¿Cuál fue el alcance del compromiso?
    c. ¿Tuvieron éxito? ¿Por qué o por qué no?
        i. ¿Cuántas personas/encuestados aproximadamente participaron en la estrategia discutida?

3. ¿Qué forma(s) de apoyo recibió el equipo para las estrategias de participación?
    a. ¿Qué formas de apoyo habrían permitido mejorar la participación?
        i. El grado en que se priorizó/valoró el compromiso
        ii. Si había (o no) un equipo dedicado centrado en el compromiso
        iii. Costos asociados
        iv. ¿Hubo honorarios/otros incentivos para participar en las estrategias de participación?

4. ¿Podría contarme (más) sobre el equipo responsable del compromiso?
    a. ¿Qué tipos de liderazgo y habilidades están presentes o faltan?

5. Una vez que se recibió la retroalimentación del ciudadano/parte interesada/público, ¿cómo se implementó/consideró en el desarrollo del Plan Nacional de Calidad de la Atención?

Mirando hacia el futuro

1. ¿Podría compartir algunas oportunidades para mejorar la participación del público en el futuro?

2. Para los intereses que no fueron identificados/abordados (perspectivas faltantes de ciudadanos/partes interesadas), ¿cómo se podría involucrar mejor a estos grupos y abordar mejor sus intereses en el futuro?

3. ¿Qué diferencia marcó la participación del público y la comunidad en el desarrollo de la estrategia nacional de calidad de atención de Colombia?

4. En su opinión, ¿de qué manera fue efectiva la participación general?
    a. ¿De quién son los intereses (ciudadanos/partes interesadas/público) que se han identificado y reconocido?

Evolución de la estrategia de Calidad de Atención de Colombia

1. Al comparar los planes nacionales de calidad de atención (2014 versus 2021), vemos que ha habido más referencias de compromiso en el plan de 2021. ¿Podrías contarme un poco sobre cómo se produjo este cambio?
    a. ¿Quién participó en el desarrollo de ambas estrategias? ¿Cómo cambiaron los equipos?
    b. ¿Hubo críticas a (la falta de) estrategias de participación en el plan de 2014?
        i. En caso afirmativo, ¿cómo se abordaron (o no) estos aspectos en el plan actualizado?

Preguntas finales

1. ¿Cómo considera usted una participación significativa del público y de la comunidad en el cambio de los sistemas de salud?

2. ¿Qué cree que otros contextos/países podrían aprender de los esfuerzos de Colombia?

3. ¿Conoce otros contextos/países con ejemplos sólidos de participación ciudadana/actor/público en el desarrollo de estrategias de calidad de atención?
